# Overexpression of miR-21 in stem cells improves ovarian structure and function in rats with chemotherapy-induced ovarian damage by targeting PDCD4 and PTEN to inhibit granulosa cell apoptosis

**DOI:** 10.1186/s13287-017-0641-z

**Published:** 2017-08-14

**Authors:** Xiafei Fu, Yuanli He, Xuefeng Wang, Dongxian Peng, Xiaoying Chen, Xinran Li, Qing Wang

**Affiliations:** Department of Obstetrics and Gynecology, Zhujiang Hospital, Southern Medical University, Guangzhou, Guangdong People’s Republic of China

**Keywords:** miR-21, Bone marrow derived mesenchymal stem cells, Chemotherapy-induced premature ovarian failure, Apoptosis, PTEN, PDCD4

## Abstract

**Background:**

Chemotherapy-induced premature ovarian failure (POF) is a severe complication affecting tumor patients at a childbearing age. Mesenchymal stem cells (MSCs) can partially restore the ovarian structure and function damaged by chemotherapy. miR-21 is a microRNA that can regulate cell apoptosis. This study discusses the repair effect and mechanism of MSCs overexpressing miR-21 on chemotherapy-induced POF.

**Methods:**

Rat MSCs and granulosa cells (GCs) were isolated in vitro. MSCs were transfected with miR-21 lentiviral vector (LV-miR-21) to obtain MSCs stably expressing miR-21 (miR-21-MSCs). The microenvironment of an ovary receiving chemotherapy was mimicked by adding phosphamide mustard (PM) into the cellular culture medium. The apoptosis rate and the mRNA and protein expression of target genes PTEN and PDCD4 were detected in MSCs. Apoptosis was induced by adding PM into the culture medium for GCs, which were cocultured with miR-21-MSCs. The apoptosis rate and the mRNA and protein expression of PTEN and PDCD4 were detected. The chemotherapy-induced POF model was built into rats by intraperitoneal cyclophosphamide injection. miR-21-MSCs were transplanted into the bilateral ovary. The rats were sacrificed at 15, 30, 45, and 60 days after the last injection. The ovarian weights, follicle count, estrous cycle, and sex hormone levels (estradiol (E2) and follicle-stimulating hormone (FSH)) were detected. Apoptosis of GCs was determined by TUNEL assay. The miR-21 and mRNA and protein expression of PTEN and PDCD4 were determined.

**Results:**

The apoptosis decreased in MSCs transfected with miR-21. The mRNA and protein expression of target genes PTEN and PDCD4 was downregulated. GCs cocultured with miR-21-MSCs showed a decreased apoptosis, an upregulation of miR-21, and a downregulation of PTEN and PDCD4. Following the injection of miR-21-MSCs, the ovarian weight and follicle counts increased; E_2_ levels increased while FSH levels decreased, with less severe apoptosis of GCs. The miR-21 expression in the ovaries was upregulated, while the mRNA expression and protein expression of PTEN and PDCD4 were downregulated.

**Conclusions:**

Overexpression of miR-21 in MSCs promoted efficacy against chemotherapy-induced POF and its improvement of the repair effect was related to the inhibition of GC apoptosis by targeting PTEN and PDCD4.

## Background

Premature ovarian failure (POF) is a gynecological endocrine disease with a decrease in estrogen levels and gonadotropin, which manifests as irregular menstruation, amenorrhoea, infertility, and perimenopause syndrome affecting women before the age of 40 years. This condition can bring about an adverse impact on reproductive, psychological, and physical health [1]. The main causes of POF include genetics, immunity, and iatrogenic (chemotherapy and radiotherapy) and other idiopathic factors [2]. With the research and development and application of chemotherapeutic agents, the survival of tumor patients has been increasing. Correspondingly, the incidence of chemotherapy-induced POF is growing as well, and this condition is a challenge for both young cancer patients and physicians [3]. However, no radical cure is yet available for reversing the chemotherapy-induced damage to the ovarian structure and function.

Mesenchymal stem cells (MSCs) are somatic stem cells with multilineage differentiation potential and are considered the most promising seeding cells for stem cell therapy due to the ease of isolation and culture and amplification [4]. Reports show that bone marrow-derived MSCs can repair spinal, myocardial, and skin damage [5, 6]. Our preliminary research indicates that bone marrow-derived MSCs could inhibit the apoptosis of granulosa cells (GCs) and partially repair the chemotherapy-induced damage to the ovarian structure and function [7]. However, some transplanted bone marrow MSCs died and the repair effect was not so satisfactory.

MicroRNAs (miRNAs) are noncoding single-stranded RNAs shorter than 22 nucleotides in eukaryotes. They can bind to the base sequences of the 3’-untranslated region (UTR) of the target mRNA, regulating the cleavage or translation of target mRNA. miRNAs play regulatory roles in a series of physiological and pathological processes such as cell proliferation, apoptosis, differentiation, and pathogen infection [8]. miR-21 is among the earliest discovered miRNAs in mammals. Study shows that miR-21 is related to apoptotic regulation in many cells [9–11]. miR-21 is also regulatory for the apoptosis of GCs and follicular development [12]. However, few reports have been concerned with whether miR-21 enhances the repair effect of MSCs in chemotherapy-induced POF.

In this study, we constructed the miR-21 lentiviral vector to transfect the MSCs; the resulting MSCs were denoted as miR-21-MSCs. We discuss the effect of miR-21 overexpression on the apoptosis of MSCs in the chemotherapy-induced ovarian microenvironment. The miR-21-MSCs were cocultured with the GCs whose apoptosis was induced by a chemotherapeutic agent. The apoptosis rate was measured. The miR-21-MSCs were transplanted to the ovaries of a rat model in vitro. The repair effect of MSCs overexpressing miR-21 in chemotherapy-induced POF and the repair mechanism are discussed.

## Methods

### Laboratory animals

This study was performed in strict accordance with the recommendations of the Guide for the Care and Use of Laboratory Animals of the National Institutes of Health. The protocol was approved by the Committee on the Ethics of Animal Experiments of Southern Medical University (permit number L2015035). All surgery was performed under sodium pentobarbital anesthesia, and every effort was made to minimize suffering.

Female clean-grade Wistar rats weighing 180–200 g were provided by the laboratory animal center of Southern Medical University. The rats were reared at room temperature of 23 ± 2 °C, humidity of 45–55%, and light duration of 12 h. The rats were allowed free access to water and acclimatized for 3–5 days. The vaginal smears were prepared before the formal experiment, and rats with a normal estrous cycle were included. The baseline levels of the sex hormones estradiol (E_2_) and follicle-stimulating hormone (FSH) were determined by collecting 1 ml of blood from the tail vein.

### Isolation and in vitro culture of bone marrow MSCs

Bone marrow MSCs were isolated by density gradient centrifugation. The rats were anesthetized to harvest the tibia and femur, which were made into a single-cell suspension. The suspension was loaded into a centrifuge tube containing Percoll solution (density 1.083). Caution was taken not to mix the two liquids together, and the volume ratio of the two liquids was 1:1. The cells were centrifuged at 2500 rpm for 20 min. The milky white intermediate layer of mononuclear cells was collected, washed with phosphate-buffered saline (PBS) twice and suspended in complete culture medium (DMEM/F12 containing 10% fetal bovine serum). The cells were inoculated to the plate at 1 × 10^6^ cells/ml, and MSCs of the second to fourth generation were harvested for further experiments. The cellular expressions of CD29, CD34, CD44, and CD45 were detected using a flow cytometer.

### Isolation and in vitro culture of GCs

Pregnant mare serum gonadotropin (PMSG) was injected subcutaneously into female Wistar rats aged 3–4 weeks at a dose of 60 IU/rat. The rats were sacrificed 48 h later and the ovaries were harvested. The mature follicles on the surface were pierced with a syringe under the dissecting microscope so that the GCs were released into the culture medium and then isolated. GCs were washed with PBS twice and suspended in culture medium for 24 h.

### Construction of the miR-21 lentiviral vector (LV-miR-21)

The miR-21 lentiviral vector was constructed. The lentiviral expression vector pLVX-shRNA2 was provided by Clontech (USA; vector no. VT1457). Polymerase chain reaction (PCR) was performed using DNA containing the rno-miR-21-5p sequence as the template. The amplification products were isolated and purified using 1% agarose gel electrophoresis. The target fragments were retrieved and double digested with BamHI and EcoRI. After further purification, the rno-miR-21-5p fragment was obtained. Empty pLVX-shRNA2 vector was double digested with BamHI and EcoRI. The linearized vector was retrieved and ligated to the purified PCR products in the presence of T4DNA ligase at 16 °C overnight. The *E.coli* DH5α competent cells were transformed with the ligation product and evenly applied to the LB plate containing ampicillinum. The cells were incubated at 37 °C overnight. A few colonies were picked and dissolved in LB medium. PCR amplification was performed using 1 μl of these colonies as the template. The PCR products were verified by agarose gel electrophoresis. Positive clones were cultured and the plasmid was extracted and sequenced by Shanghai Invitrogen Biotech Co., Ltd. The lentiviral vectors were packaged and the titer was determined as 1 × 10^9^/ml.

### Transfection of MSCs with LV-miR-21

The lentiviral vectors carrying the miR-21 gene were added into the MSCs at a multiplicity of infection (MOI) of 20. After transfection for 24–48 h, the expression of the green fluorescent protein was observed under the fluorescence microscope. The transfection efficiency was calculated.

Three groups were set up: the MSC group (no transfection with the lentiviral vectors), the LV group (transfection with the empty vectors), and the miR-21 group (transfection of MSCs with the miR-21 lentiviral vector at MOI of 20). The miR-21 level was determined using quantitative reverse-transcription PCR (qRT-PCR) in each group.

### Effects of miR-21 overexpression on the apoptosis of MSCs in the local microenvironment of ovaries damaged by chemotherapy

Phosphamide mustard (PM) is the active product of metabolism of cyclophosphamide (CTX) and proves toxic to the ovaries [13]. Therefore, for the in vitro experiment, we used PM instead of CTX. Three groups were set up: the MSC group, the LV group, and the miR-21 group. No treatment was given in the MSC group; MSCs in the LV group and miR-21 group were transfected with empty LV and LV-miR-21, respectively, followed by the addition of 30 μmol/L PM to mimic the local microenvironment of ovaries damaged by chemotherapy. The apoptotic rate of MSCs was detected with a flow cytometer. mRNA expression of PTEN and PDCD4 was determined using qRT-PCR; protein expression of these two genes was determined using Western blotting.

### Effects of MSCs overexpressing miR-21 on the apoptosis of GCs

Five groups were set up: the normal group, the PM group, the miR-21 group, the MSC group, and the miR-21-MSC group. Cells in the normal group were not treated with PM; cells in the PM group had 30 μmol/L PM added to induce apoptosis; for the miR-21 group, the apoptosis was induced by adding PM and then the cells were transfected with LV-miR-21; for the MSC group and miR-21-MSC group, the cells were treated with PM and were then respectively transfected with MSCs and miR-21-MSCs at a 1:1 proportion. The apoptosis of GCs was detected after 48 h with a flow cytometer. The mRNA expression of miR-21, PTEN, and PDCD4 was determined using qRT-PCR; the protein expression was determined using Western blotting.

### Construction of rat models of chemotherapy-induced POF

Rat models of chemotherapy-induced POF were constructed by intraperitoneal injection of CTX. The first injection was performed at a dose of 50 mg/kg, which was followed by continuous injection at a dose of 8 mg/kg for 14 days [7].

### Transplantation of miR-21-MSCs and post-transplantation observation

The rats were randomly divided into five groups, with 20 rats in each group. The five groups were: the normal group, the model group, the miR-21 group, the MSC group, and the miR-21-MSC group. Chemotherapy-induced POF models were built into the last four groups. On the day of the last CTX injection 1 × 10^6^ LV-miR-21 were injected into the bilateral ovaries in the miR-21 group; for the MSC group, 1 × 10^6^ MSCs were injected into the bilateral ovaries; for the miR-21-MSC group, 1 × 10^6^ miR-21-MSCs were injected under chloral hydrate anesthesia.

The estrous cycle was determined by preparing Pap smears at 08:00 every day after transplantation. Blood samples were collected during the estrous cycle at 15, 30, 45, and 60 days after transplantation and stored at –80 °C. E_2_ and FSH levels were determined in these blood samples. Five rats were sacrificed in each group at 15, 30, 45, and 60 days after transplantation. The bilateral ovaries were harvested. One ovary was used to determine the weight, structure, follicle counts, and apoptosis of GCs; the other ovary was used to determine the expression of miR-21 as well as mRNA and protein expression of PTEN and PDCD4.

### Determination of E_2_ and FSH levels

FSH levels were determined using a radioimmunoassay kit provided by Beijing North TZ-Biotech Develop. Co., Ltd. E_2_ levels were determined using the chemiluminescence method.

### Ovarian weight, follicle counts, and ovarian morphology

Ovaries were harvested and the adipose tissues were removed. The ovaries were weighed and their structures were observed with the naked eye. The tissues were embedded in paraffin, sliced into 5-μm thick segments and subjected to hematoxylin and eosin (H&E) staining. The ovarian structure was observed under the microscope, and the follicle counts were determined by reference to the literature [14]. The twelfth section was taken to determine the follicle counts for each sample. Primordial follicles only contained single-layer spindle-shaped GCs; the single layer of GCs in the primary follicles had at least three columnar GCs; secondary follicles contained at least two layers of GCs; and antral follicles had at least two layers of GCs with follicular cavities.

### qRT-PCR

Total RNA was extracted from MSCs, GCs, and ovary tissues using Trizol® reagent (Invitrogen, Life Technologies, Carlsbad, CA, USA) according to the manufacturer's protocol. The complementary DNA (cDNA) was synthesized from isolated RNA samples with a Mir-X miRNA First-Strand Synthesis Kit (TaKaRa Bio Inc., Shiga, Japan). The expression levels of miR-21, PTEN, and PDCD4 were assessed by real-time qRT-PCR using a Mir-X miRNA qRT-PCR SYBR Kit (TaKaRa, Japan) and an ABI 7500 real-time PCR system. All procedures were performed following the manufacturer’s instructions. The relative fold changes of expression were analyzed according to the quantitative-comparative (Ct) method and were normalized to the expression level of U6 small nuclear RNA. GAPDH was used as an internal control. The analysis of each sample was repeated in triplicate for both the target and the reference gene. The primer sequences were 5’-TAGCTTATCAGACTGATGTTG-3’ and 5’-GCTGTCAACGATACGCTACGTAACG-3’ for miR-21, 5′-CCCAGTTTGTGGTCTGCCAGC-3′ and 5′-ATGAGCTTGTCCTCCCGCCG-3′ for PTEN, and 5'-TTGAGCACGGAGATACGAAC-3' and 5'-GTCCCGCAAAGGTCAGAAAG-3' for PDCD4.

### Western blot

Nuclear and cytoplasmic protein-enriched lysate fractions were isolated from homogenized MSCs, GCs, and ovary tissues using cell lysis solution. The protein concentration was quantified using the Lowry method according to the manufacturer’s instructions. The protein fractions (10–20 μg per lane) were subjected to 12% sodium dodecyl sulfate-polyacrylamide gel electrophoresis (SDS-PAGE) and transferred onto hybrid polyvinylidene fluoride (PVDF) membranes (Millipore, Bedford, MA, USA). After blocking with 5% (w/v) nonfat dry milk in TBST (Tris-buffered saline containing 50 mM Tris/HCl, pH 8.8, 125 mM NaCl, and 0.05% Tween-20), the PVDF membranes were washed four times (15 min each) with TBST at room temperature, immunoblotted overnight at 4 °C with specific monoclonal antibodies including rabbit anti-PDCD4 (1:1000; PDCD4 (D29C6) XP® Rabbit mAb, Cell Signaling Technology, Inc., Danvers, MA, USA) and rabbit anti-PTEN (1:1000; PTEN (D4.3) XP® Rabbit mAb, Cell Signaling Technology, Inc.), and then immunoblotted with FITC-conjugated goat anti-rabbit IgG antibody (1:1000; Beyotime Institute of Biotechnology, Jiangsu, China) at room temperature for 26 min. The immunoreactivity was visualized using an ECL kit (Perkin-Elmer Life Science, Fremont, CA, USA) and the band density analysis of the blots was performed with a gel imaging system (Kodak, 4000R PRO, Rochester, NY, USA). β-Actin (Beyotime Institute of Biotechnology) was used as an internal reference for normalization. All steps were repeated in triplicate.

### TUNEL apoptosis detection of GCs

The apoptosis of GCs was determined using the Apop Tag kit (Nanjing KeyGen Biotech Co., Ltd.). For each sample, eight fields of view were randomly selected. The apoptosis index was estimated for 100 GCs by dividing the number of apoptotic GCs by the total number of GCs.

### Statistical analysis

Data are reported as mean ± standard deviation and analyzed statistically using the SPSS 20.0 software. An independent sample *t* test was used for comparing the means between two groups; one-way analysis of variance (ANOVA) was used for multiple comparison among three or more groups. Intergroup comparisons were performed by the SNK method. A *P* value less than 0.05 was considered to denote a significant difference.

## Results

### Culture and identification of MSCs

**Fig. 1:**
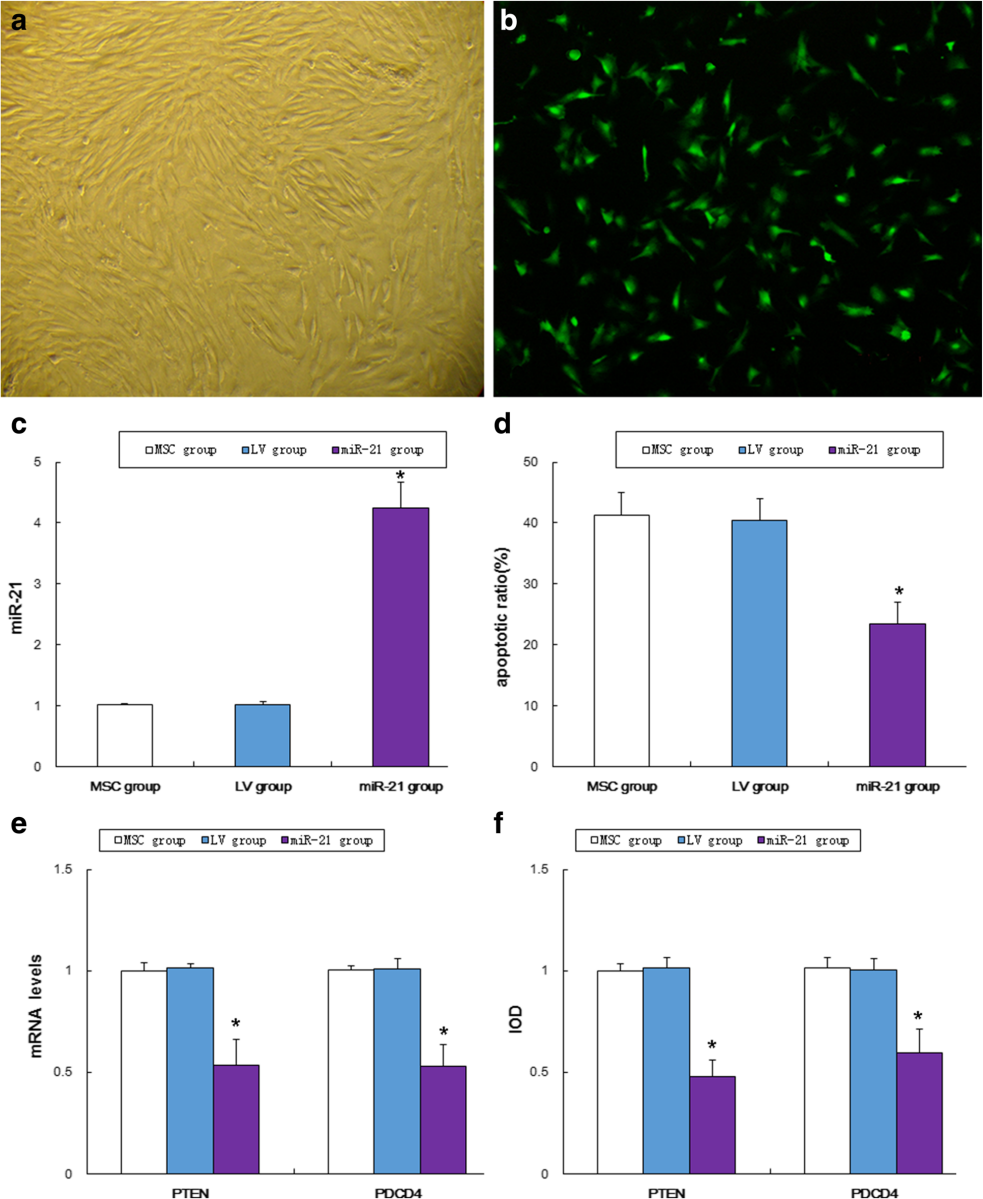
Apoptosis of MSCs and expression of miR-21, PTEN, and PDCD4. **a** Mesenchymal stem cells (*MSCs*) of the third generation (×100). **b** MSCs transfected with LV-miR-21 at a MOI of 20, emitting green fluorescence under the fluorescence microscope (×100). **c** Expression of miR-21 in MSCs transfected with LV-miR-21. *P < 0.05, compared to the MSC group and the LV group. **d** Comparison of apoptotic rates between the groups. *P < 0.05, compared to the MSC group and the LV group. **e** Comparison of mRNA expression of PTEN and PDCD4 between the groups. *P < 0.05, compared to the MSC group and the LV group. **f** Comparison of protein expression of PTEN and PDCD4. *P < 0.05, compared to the MSC group and the LV group

MSCs of the third generation had uniform morphology as fibroblast-like long spindles and showed an orderly arrangement (Fig. 1a). Flow cytometry indicated that over 90% of MSCs were negative for CD34 and CD45 and positive for CD44 and CD29. This agrees with previous findings [15]. The cultured cells were MSCs and not hematopoietic stem cells.

### Construction of miR-21 lentiviral vector and transfection of MSCs

The miR-21 lentiviral vector pLVX-shRNA2-miR-21-5p was constructed and double digested with EcoRI and BamHI. The fragments were identified by agarose gel electrophoresis and found to be 7888 bp and 117 bp, respectively. Sequencing the positive clones indicated that the rno-miR-21-5p sequence inserted into the recombinant plasmid was identical to the target sequence. Thus, the miR-21 lentiviral vector was successfully constructed.

MSCs were transfected with miR-21 lentiviral vectors at a MOI of 20. The expression of green fluorescence protein was observed under the fluorescence microscope (Fig. 1b). The transfection efficiency was 92.20% ± 1.96%. qRT-PCR indicated that miR-21 expression of the MSC group, the LV group, and the miR-21 group was 1.0154 ± 0.0258, 1.0191 ± 0.0500, and 4.2410 ± 0.4367, respectively. A significant difference was found among the three groups according to one-way ANOVA (*F* = 268.032, *P* = 0.000). Intergroup comparisons using the SNK method indicated no statistical difference between the MSC group and the LV group; the expression of the miR-21 group was higher than that of the MSC group and the LV group (Fig. 1c).

### Effects of miR-21 overexpression on apoptosis of MSCs and expression of PTEN and PDCD4

The apoptotic rate of MSCs was determined with a flow cytometer. The apoptotic rate of the MSC group, the LV group, and the miR-21 group was 41.35 ± 3.63%, 40.34 ± 3.59%, and 23.49 ± 3.61%, respectively; the three groups were significantly different. The apoptotic rate of the miR-21 group was lower than that of the MSC group and the LV group (*F* = 38.597, *P* = 0.000; Fig. 1d). Thus, miR-21 overexpression inhibited the chemotherapy-induced apoptosis of MSCs. The mRNA expression and protein expression of PTEN and PDCD4 were downregulated in the miR-21 group as compared with the MSC group and the LV group (Fig. 1e and f).

### miR-21 overexpression in MSCs inhibited apoptosis of GCs

**Fig. 2:**
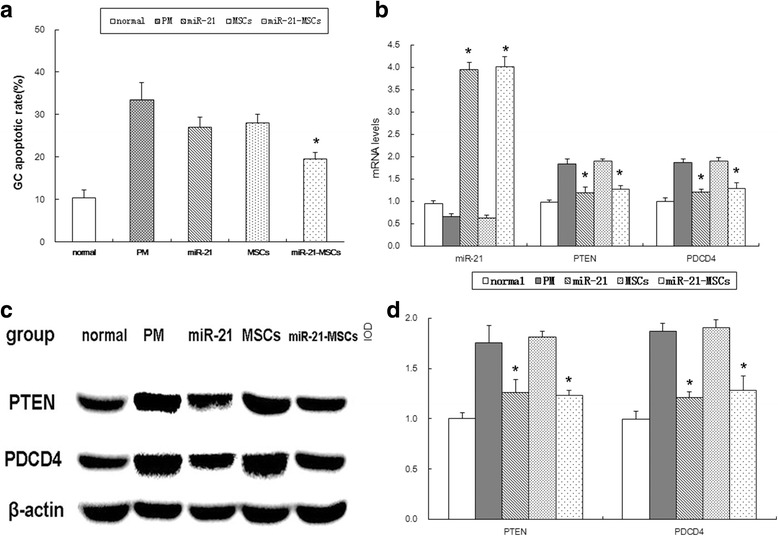
Apoptosis of GCs and expression of miR-21, PTEN, and PDCD4. **a** Comparison of apoptotic rates of granulosa cells (*GCs*) between the groups. *P < 0.05, versus the miR-21 group and the mesenchymal stem cell (*MSC*) group. **b** miR-21 expression of GCs, and comparison of mRNA expression of PTEN and PDCD4.*P < 0.05, versus the MSC group. **c**, **d** Western blot detection of PTEN and PDCD protein expression (**c**) with quantification (**d**). *P < 0.05, compared to the MSC group

According to the results of flow cytometry, the apoptotic rate of the normal group, the PM group, the miR-21 group, the MSC group, and the miR-21-MSC group was 10.4 ± 1.82%, 33.4 ± 4.22%, 27 ± 2.45%, 28 ± 2%, and 19.6 ± 1.52%, respectively; the apoptotic rate of the five groups differed significantly (*F* = 59.35, *P* = 0.00), and the apoptotic rate of the miR-21-MSC group was lower than that of the miR-21 group and the MSC group, but higher than that of the normal group (Fig. 2a).

### Expression of miR-21 and its target genes in GCs

The miR-21 expression in the miR-21 group and the miR-21-MSC group was upregulated significantly compared with the other groups; the mRNA expression and protein expression of PTEN and PDCD4 were downregulated significantly compared with the PM group and the MSC group (Fig. 2b–d).

### Pap smears

The estrous cycle was normal in the normal group, whereas there was estrous cycle disturbance in the model group, the miR-21 group, the MSC group, and the miR-21-MSC group after the last injection. For the latter, the estrous cycle was delayed, absent, or persisted. There were 6 rats in the miR-21 group, 6 rats in the MSC group, and 10 rats in the miR-21-MSC group which had a restored normal estrous cycle at 16–30 days after the last injection. There were 3, 2, and 5 rats, respectively, in these groups with restored normal estrous cycle at 31–45 days after the last injection, and there were 2, 2, and 4 rats, respectively, with restored normal estrous cycle at 46–60 days after the last injection. However, estrous cycle disturbance persisted in the model group (Table 1).

**Table 1:** Number of rats with a normal estrous cycle in the five groups

Group	1–15 days	16–30 days	31–45 days	46–60 days
Normal group	20	15	10	5
Model group	0	0	0	0
miR-21 group	0	6	3	2
MSC group	0	6	2	2
miR-21-MSCs group	0	10	5	4

*MSC* mesenchymal stem cell

### Ovarian appearance, weight, and follicle counts

**Fig. 3:**
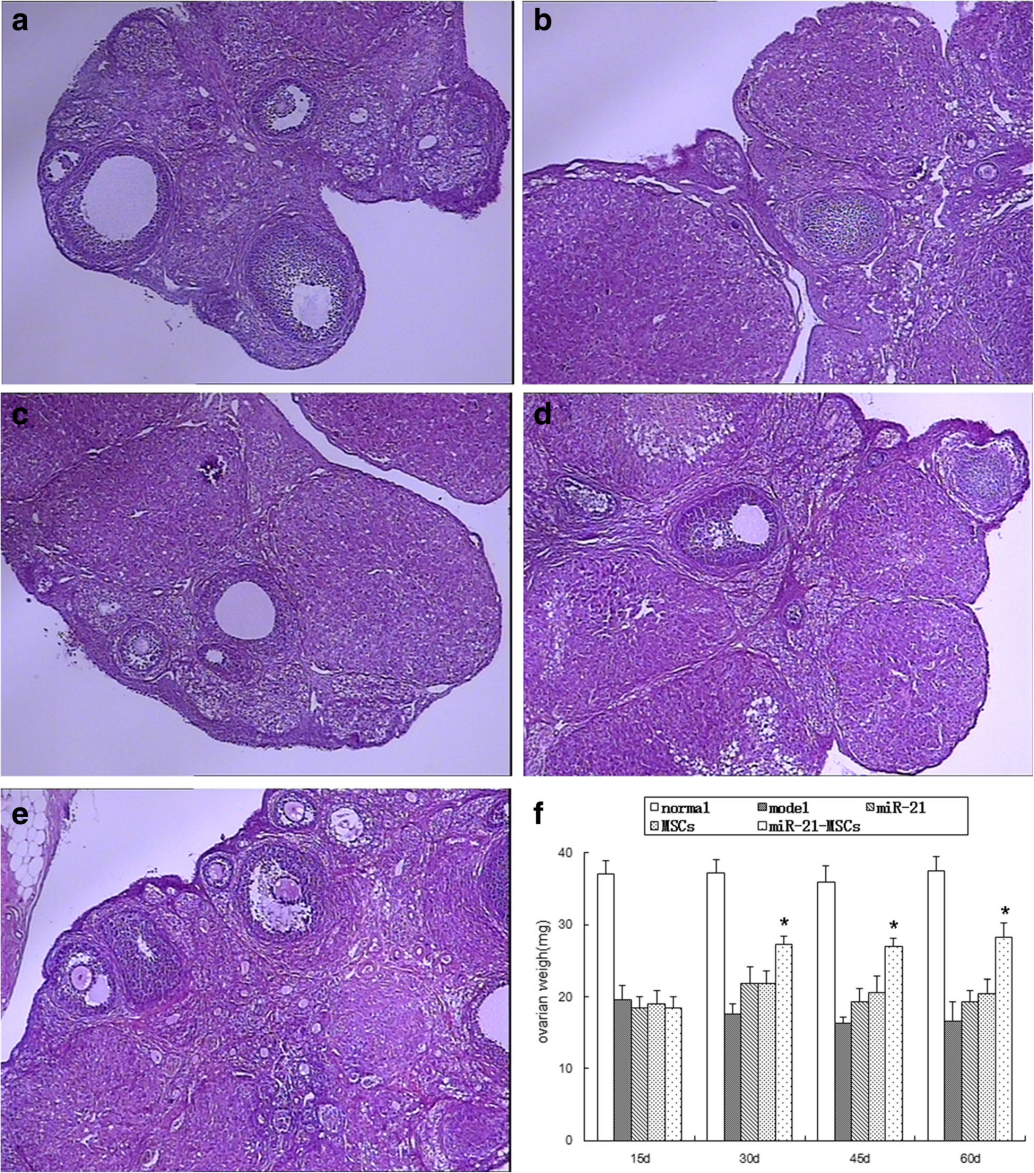
Ovarian weight and structure. **a** Ovarian structure at 45 days after injection in the normal group, with the observation of follicles at different developmental stages and corpora lutea. **b** Ovarian structure at 45 days after injection in the model group, with a dramatic reduction in follicle counts. **c** Ovarian structure at 45 days after injection in the miR-21 group, with an increase in follicle counts as compared with the model group. **d** Ovarian structure at 45 days after injection in the mesenchymal stem cell (*MSC*) group, with an increase in follicle counts as compared with the model group but comparable to that in the miR-21 group. **e** Ovarian structure at 45 days after injection in the miR-21-MSC group, with an increase in follicle counts as compared with the miR-21 group and MSC group. **f** Ovarian weight at 15, 30, 45, and 60 days after injection in each group. *P < 0.05, compared to the miR-21 group and the MSC group

The ovarian appearance was normal in the normal group, showing a red color and several white raised spots on the surface. At 15 days after injection the ovarian size shrank in the miR-21 group, the MSC group, and the miR-21-MSC group; the ovaries were pale white with fewer raised spots on the surface. At 30, 45, and 60 days after injection there were no apparent changes in ovarian appearance in the model group. The ovaries were enlarged in the miR-21 group, the MSC group, and the miR-21-MSC group at 30, 45, and 60 days after injection, with more raised spots on the surface. As compared with the other three groups, there were more raised spots on the surface in the miR-21-MSC group. H&E staining revealed follicles at different developmental stages and several corpora lutea in the normal group. Several layers of GCs were found inside the follicles. After the last injection there was a reduction in the number of growing follicles as well as corpora lutea in the model group; ovarian angiogenesis, proliferation of fibrous tissues, thickening of vascular walls, and hyaline degeneration were observed. At 45 and 60 days after injection there were more follicles in the miR-21 group, the MSC group, and the miR-21-MSC group than in the model group (Fig. 3a–e).

The ovarian weight at 15 days after injection was 37.12 ± 1.74 mg in the normal group, which was higher than that of the model group (19.35 ± 2.01 mg) and the miR-21 group (18.41 ± 1.66 mg); ovarian weight was 19.04 ± 1.27 mg and 18.38 ± 1.61 mg in the MSC group and the miR-21-MSC group, respectively. There was no significant difference among the last four groups. At 30, 45, and 60 days after injection the ovarian weight increased in the MSC group and the miR-21-MSC group, and it was higher than that in the model group. The ovarian weight increased more considerably in the miR-21-MSC group and exceeded that of the miR-21 group and the MSC group (Fig. 3f).

**Fig. 4:**
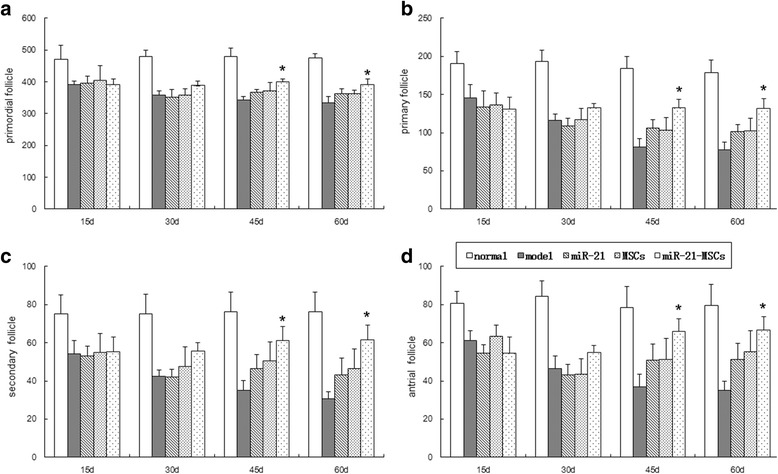
Comparison of follicle counts at 15, 30, 45, and 60 days after injection among the groups. **a** Count of primordial follicles. **b** Count of primary follicles. **c** Count of secondary follicles. **d** Count of antral follicles. *P < 0.05, compared to the miR-21 group and the mesenchymal stem cell (*MSC*) group

At 15 days after injection the follicle counts of the normal group were much higher than those of the model group, the miR-21 group, the MSC group, and the miR-21-MSC group; there was no significant difference among the last four groups. At 30 days after injection the follicle counts in the miR-21-MSC group increased as compared with the model group, the miR-21 group, and the MSC group; however, there was no significant difference among the last three groups. At 45 and 60 days the follicle counts of the model group further decreased, and those of the miR-21 group, the MSC group, and the miR-21-MSC group were all higher than that of the model group. Follicle counts of the miR-21-MSC group were higher than those of the miR-21 group and the MSC group. However, the follicle counts of the miR-21-MSC group were still lower than those of the blank control group (Fig. 4).

### Sex hormone levels

**Fig. 5:**
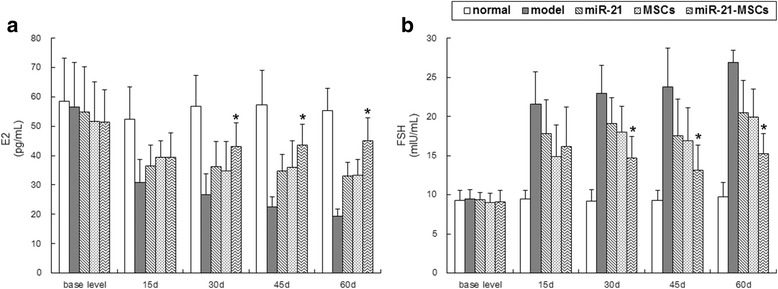
Comparison of sex hormone levels among the five groups. **a** Estradiol (*E*_*2*_) levels at 15, 30, 45, and 60 days after injection in each group. *P < 0.05, compared to the miR-21 group and the mesenchymal stem cell (*MSC*) group. **b** Follicle-stimulating hormone (*FSH*) levels at 15, 30, 45, and 60 days after injection in each group. *P < 0.05, compared to the miR-21 group and the MSC group

The baseline levels of E_2_ and FSH were not significantly different between the groups. At 15 days after injection there was significant difference in the E_2_ and FSH levels among the five groups (*F*1 = 18.567, *P*1 = 0.000; *F*2 = 24.991, *P*2 = 0.000). The E_2_ levels in the model group, the miR-21 group, the MSC group, and the miR-21-MSC group declined, while the FSH levels increased. The E_2_ levels of the miR-21 group, the MSC group, and the miR-21-MSC group were higher than that of the model group, while the FSH levels of the former groups were lower than that of the model group; there was no significant difference among the former three groups. At 30, 45, and 60 days after injection the sex hormone levels of the five groups differed considerably, with the normal group maintaining the baseline levels. The E_2_ levels of the model group declined continuously, while FSH levels increased continuously. The changes in the miR-21 group and the MSC group were milder, and there was no significant difference between these two groups. The sex hormone levels of the miR-21-MSC group further stabilized, showing significant differences as compared with the miR-21 group and the MSC group, but not reaching the level of the normal group (Fig. 5).

### Apoptosis of GCs

**Fig. 6:**
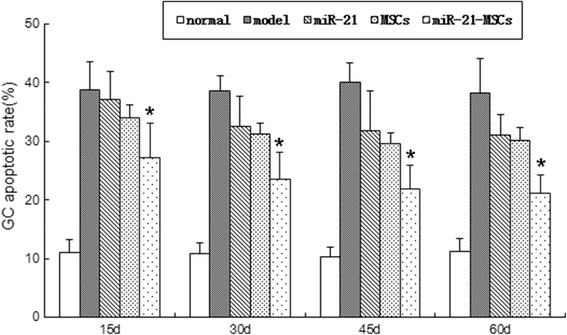
Apoptosis of GCs in each group. Apoptotic rate of granulosa cells (*GCs*) at 15, 30, 45, and 60 days after injection in each group. *P < 0.05, versus the miR-21 group and the mesenchymal stem cell (*MSC*) group

There was a significant difference in the apoptotic rate of GCs at 15 days after injection among the five groups. The apoptotic rate was 11% ± 2.34% in the normal group, which was lower than that in the model group (38.8 ± 4.66%), the miR-21 group (37.2 ± 4.60%), the MSC group (34 ± 4.90%), and the miR-21-MSC group (27.2 ± 5.89%). Comparison among the latter four groups indicated a higher level in the miR-21-MSC group than in the model group, the miR-21 group, and the MSC group; however, there was no significant difference between the model group, the miR-21 group, and the MSC group. At 30, 45, and 60 days after injection the apoptotic rate of GCs in the miR-21-MSC group further decreased, and it was much lower than that of the model group, the miR-21 group, and the MSC group, but still higher than that of the normal group (Fig. 6).

### Expression of miR-21 and target genes PTEN and PDCD4 in the ovarian tissues

**Fig. 7:**
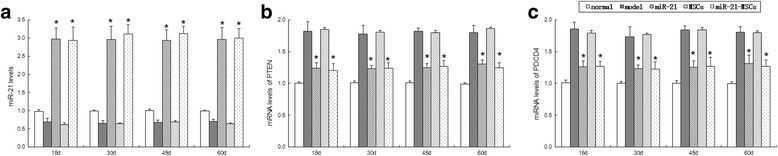
Comparison of miR-21 and mRNA expression of PTEN and PDCD4 in ovarian tissues between the groups. Expression of miR-21 (**a**) and PTEN (**b**) and PDCD4 (**c**) mRNA was detected using qRT-PCR. *P < 0.05, compared to the model group and the mesenchymal stem cell (*MSC*) group

The miR-21 expression in the miR-21 group and the miR-21-MSC group was significantly higher than that of the normal group, the model group, and the MSC group at different time points after injection. There was no difference between the miR-21 group and the miR-21-MSC group. Comparisons showed that miR-21 expression was higher in the normal group than in the model group and the MSC group, but it was not significantly different between the model group and the MSC group (Fig. 7).

**Fig. 8:**
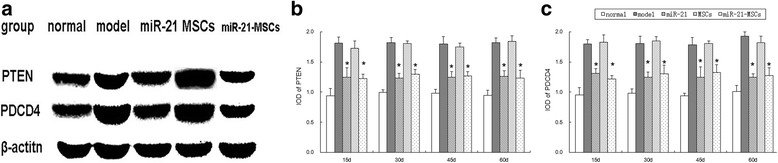
Protein expression of PTEN and PDCD4 in the ovarian tissues in each group. **a** Protein expression of PTEN and PDCD4 was determined by Western blot, with **b**, **c** quantification. *P < 0.05, compared to the model group and the mesenchymal stem cell (*MSC*) group

At different time points after injection the mRNA and protein expression of PTEN and PDCD4 was significantly different among the five groups. The expression in the miR-21 group and the miR-21-MSC group was lower than that of the model group and the MSC group, but higher than the normal group. There was no significant difference between the model group and the MSC group or between the miR-21 group and the miR-21-MSC group (Fig. 8).

## Discussion

A reduction in estrogen levels and reproductivity caused by POF poses a great threat to the reproductive health and quality of life for women of childbearing age [16]. Chemotherapy-induced POF is gaining increased attention among all the causes of POF since chemotherapy is now widely used to treat malignancies and immune diseases. Along with technical progress, chemotherapy has greatly increased the survival of cancer patients. For young female patients, POF is a severe short- and long-term complication that affects their lives and that receives academic attention. The mechanism of chemotherapeutic agents inducing ovarian follicle atresia remains unclear. It is generally believed that the chemotherapeutic agents have an adverse impact on the growth, development, and maturity of follicles, resulting in ovarian follicle atresia, fibrosis of ovarian tissues, and an inability of GCs to secrete estrogen and progesterone [17]. Common treatment now includes hormone therapy, gonadotropin releasing hormone agonist (GnRHa) therapy, oocyte and embryo cryopreservation and cryopreservation, and transplantation of ovarian tissues [18]. However, none of these therapies are very satisfactory.

MSCs are known for their self-renewal and multilineage differentiation potential as well as the prospect of their application in cellular and genetic therapies [19]. The efficacy of transplantation of MSCs has been verified in animal models of different diseases [5, 6]. MSCs also offer hope in the treatment of POF in animal models. We found through preliminary studies that MSCs secreted a variety of cytokines in vitro, including vascular endothelial growth factor (VEGF), insulin-like growth factor-1 (IGF-1), and hepatocyte growth factor (HGF). MSCs can inhibit chemotherapy-induced apoptosis of GCs by upregulating Bcl-2 protein and other cytokines. In the rat model of chemotherapy-induced ovarian damage, transplantation of MSCs alleviated cell apoptosis and improved ovarian function [7]. However, the repair effect of MSCs in chemotherapy-induced POF is only partial as the apoptosis of the transplanted MSCs impairs the outcomes [20].

miR-21 is among the earliest discovered miRNAs and is present extensively in the human body. The miR-21 gene is first transcribed into pri-miR-21 in the nuclei in the presence of RNA polymerase II, which is then modified to form the mature miR-21. Promoting cell proliferation and inhibiting apoptosis, miR-21 is highly expressed in a variety of cells and tissues [21]; it also plays a decisive role in the regulation of GC apoptosis and follicular development. Study shows that miR-21 can inhibit the apoptosis of rat GCs and increase the ovulation rate. In this study, the apoptosis of GCs was inhibited by injection of LV-miR-21 to bilateral ovaries to induce miR-21 expression. There was also an increase in follicle counts, indicating the partial repair effect of miR-21 on chemotherapy-induced POF.

We transplanted MSCs overexpressing miR-21 to treat chemotherapy-induced POF. The results showed that the apoptosis of miR-21-overexpressing MSCs decreased and the vitality of the transplanted cells increased. In vitro results indicated stronger inhibitory effects on the apoptosis of GCs. As compared with the transplantation of normal MSCs or miR-21 injection alone, the number of rats with a normal estrous cycle increased following the transplantation of MSCs overexpressing miR-21; the E_2_ level increased while the FSH level decreased significantly. The number of follicles at different developmental stages increased as well. All these results suggested the strong repair effect of transplanting MSCs with miR-21 overexpression.

miRNA can bind to the target mRNA in an incompletely complementary way. One miRNA may regulate hundreds or thousands of target genes, which constitute a mutually connected and mutually restricting regulatory network. It is reported that miR-21 has about 190 target genes [22]. PDCD4 and PTEN are the most intensively studied and they play a regulatory role in cell apoptosis. miR-21 can inhibit the hydrogen oxide-induced MSC apoptosis, probably because the downstream target gene PTEN regulates the PI3K/Akt pathway [23]. In the apoptosis of MSCs induced by hypoxia/serum-free culture, upregulating miR-21 can increase the mitochondrial membrane potential and protect the mitochondrial function. This will further inhibit the mitochondrial pathway, improve the tolerance of MSCs to hypoxia/serum-free culture, and increase the vitality of cells [24]. In this study, we found a reduction in the chemotherapy-induced apoptosis of MSCs overexpressing miR-21. This means miR-21 enhanced the resistance of MSCs to chemotherapy and increased the vitality of cells. Furthermore, PTEN and PDCD4 were downregulated in MSCs, probably due to the fact that miR-21 enhanced the vitality of MSCs. PTEN regulating the downstream PI3K pathway has an impact on the proliferation and apoptosis of GCs [25]. Downregulation of PTEN with a phosphorylation of PIP-3 will activate the protein kinase B or Akt (PKB/Akt) pathway, inducing cell growth and proliferation, and inhibiting cell apoptosis [26]. Conversely, an imbalanced PTEN expression will cause POF [27]. By binding to eukaryotic initiation factor 4E (elF43), PDCD4 can inhibit the production of complexes and thus regulate protein translation at the initial stage [28]. PDCD4 is involved in the lipopolysaccharide (LPS)-mediated apoptosis [29]. In the LPS signaling pathway, Toll-like receptor 4 (TLR4) upregulates PDCD4, which induces the expression of interleukin (IL)-10 and NF-κB downstream, thus inducing cell apoptosis [30]. The expression of PDCD4 can reduce the normal DNA-damage response and protect the cells with DNA damage, therefore inhibiting cell apoptosis [31]. Downregulating PTEN and PDCD4 will inhibit cell apoptosis. In our study, miR-21 inhibited apoptosis of GCs probably by downregulating PTEN and PDCD4.

Recently, a novel population of stem cells—very small embryonic-like stem cells (VSELs)—are found to locate in the ovary surface epithelium (OSE) in mice. Chemotherapy can result in a loss of follicular reserve, but VSELs survive [32]. This provides hope that surviving VSELs can restore the ovarian structure and function for patients with chemotherapy-induced ovarian failure. The effect of MSCs over expressing miR-21 on VSELs remains unclear. Further studies are required to show whether MSCs over expressing miR-21 can stimulate and protect VSELs.

## Conclusions

To conclude, we found that: (1) MSCs overexpressing miR-21 showed a reduction in apoptosis in vitro and an increase in vitality; (2) MSCs overexpressing miR-21 caused a downregulation of PTEN and PDCD4 in vitro, which inhibited the PM-induced apoptosis of GCs; and (3) transplantation of MSCs overexpressing miR-21 into rat ovaries damaged by chemotherapy could more effectively inhibit the apoptosis of GCs as compared with the injection of MSCs or miR-21 alone. The ovarian structure and function were repaired to a greater extent by transplantation of MSCs overexpressing miR-21. This repair effect may be mediated by PTEN and PDCD4, the target genes of miR-21.

## Abbreviations

CTX: Cyclophosphamide
E_2_: Estradiol
FSH: Follicle-stimulating hormone
GC: Granulosa cell
H&E: Hematoxylin and eosin
LPS: Lipopolysaccharide
miRNA: MicroRNA
MOI: Multiplicity of infection
MSC: Mesenchymal stem cell
PBS: Phosphate-buffered saline
PCR: Polymerase chain reaction
PDCD4: Programmed cell death 4
PM: Phosphamide mustard
POF: Premature ovarian failure
PTEN: Phosphatase and tensin homolog deleted on chromosome ten
VSEL: Very small embryonic-like stem cell
